# Dimerization and oligomerization of DNA-assembled building blocks for controlled multi-motion in high-order architectures

**DOI:** 10.1038/s41467-021-23532-y

**Published:** 2021-05-28

**Authors:** Ling Xin, Xiaoyang Duan, Na Liu

**Affiliations:** 1 2. Physics Institute, University of Stuttgart, Stuttgart, Germany; 2 Max Planck Institute for Solid State Research, Stuttgart, Germany

**Keywords:** DNA nanotechnology, Nanoscale devices, Optical materials and structures

## Abstract

In living organisms, proteins are organized prevalently through a self-association mechanism to form dimers and oligomers, which often confer new functions at the intermolecular interfaces. Despite the progress on DNA-assembled artificial systems, endeavors have been largely paid to achieve monomeric nanostructures that mimic motor proteins for a single type of motion. Here, we demonstrate a DNA-assembled building block with rotary and walking modules, which can introduce new motion through dimerization and oligomerization. The building block is a chiral system, comprising two interacting gold nanorods to perform rotation and walking, respectively. Through dimerization, two building blocks can form a dimer to yield coordinated sliding. Further oligomerization leads to higher-order structures, containing alternating rotation and sliding dimer interfaces to impose structural twisting. Our hierarchical assembly scheme offers a design blueprint to construct DNA-assembled advanced architectures with high degrees of freedom to tailor the optical responses and regulate multi-motion on the nanoscale.

## Abstract (web summary)

Creation of high-order architectures using DNA devices is of interest for increasing the complexity of synthetic systems. Here, the authors, inspired by biological oligomers, create DNA dimers and oligomers that combining rotation and walking to make high-order systems with more complex conformational changes.

## Introduction

Cellular life functions through a collection of highly-controlled dynamic processes involving the self-assembly and organization of diverse molecular building blocks. These biological constructs optimized through billions of years of evolution provide us with the inspiration to create artificial systems, which emulate the structural and functional features of their natural counterparts. In particular, oligomerization is essential in many protein-involved cellular processes^1,2^. For instance, a GTPase called dynamin is at the heart of endocytic vesicle fission. The dynamin unit is an antiparallel dimer, which can oligomerize into a helical polymer. The ratchet model proposes that GTP hydrolysis powers the relative sliding of the helical turns, giving rise to twisting of the helix and eventually membrane fission^3,4^. Such a self-association mechanism through dimerization and oligomerization not only enables new functions at the intermolecular interfaces but also elicits a wealth of structural and functional advantages. This offers a blueprint to create mimics that can collectively operate to define functions in artificial machinery.

Among different state-of-the-art techniques, DNA nanotechnology^5–17^ represents a unique tool to build bio-inspired artificial systems, taking advantage of the precision, addressability, and programmability of DNA on the nanoscale. A variety of DNA-based dynamic devices^18–21^ that mimic motor proteins^22^ in living cells has been accomplished, including walkers^23–30^, rotors^31–36^, sliders^37–40^, and assembly lines^41^. Recently, efforts to constructing bio-inspired DNA-assembled systems are taking a step forward from monomeric to high-order structures^42–53^ and from simple to complex motion^38,49,54–57^. Here, we demonstrate a DNA-assembled building block with rotary and walking modules, which can form dimeric structures to execute sliding, as well as subsequent higher-order architectures linked through dimer interfaces of the rotary and walking modules for controlled multi-motion. The monomeric building block is a chiral system^58,59^, which consists of two interacting gold nanorods (AuNRs) templated by DNA origami^60–63^. The two AuNRs can carry out the rotation and walking, respectively. Through dimerization, the walking modules are merged into a sliding module, imposing relative movements between two building blocks. Further oligomerization leads to higher-order structures with alternating dimer interfaces for collective rotation and sliding, imposing structural twisting. Our work delineates one of the rudimentary steps towards self-assembled artificial structures that imitate the collective motion of protein complexes, with the ultimate goal to build and engineer cellular mimics with fully operational structural and functional features de novo. Although it is an incredible adventure, fortunately, we will be able to look at the solutions provided by nature for every problem we encounter.

## Results

### Structural design of the UNIT

**Fig. 1:**
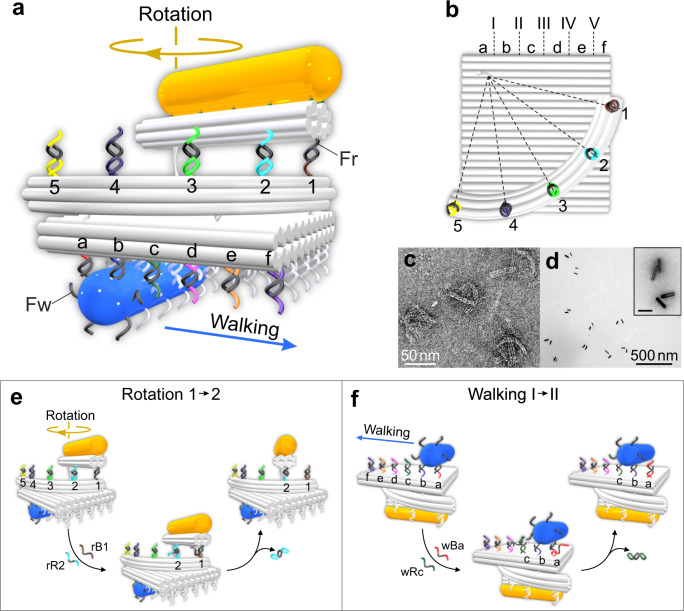
UNIT with rotary and walking modules. **a** Schematic of the UNIT. The DNA origami template consists of a double-layer plate, a 6-helix 90° arc-shaped track, and a 10-helix rotary bundle. The rotary module contains a AuNR (yellow) anchored on the rotary bundle and the arc-shaped track dressed with five rotation footholds (rfhs, rfh1–rfh5). A foot strand (Fr) is extended from one end of the rotary bundle. The walking module contains a AuNR (blue) fully decorated with foot strands for walking (Fw) and six rows of walking footholds (wfhs, wfha–wfhf). **b** Top view of the rotary (positions 1–5) and walking (positions I–V) tracks. TEM images of the DNA origami template structures (**c**) and UNITs (**d**). Inset: enlarged view of the representative UNIT structures. Scale bar: 50 nm. **e** Working principle of one-step rotation from position 1 to 2. **f** Working principle of one-step walking from position I to II. Both the stepwise rotation and walking processes are enabled by the addition of corresponding blocking and removal strands through toehold-mediated strand displacement reactions.

Figure 1a shows the schematic of the DNA-assembled building block, which is composed of a rotary module and a walking module. It is named UNIT. The DNA origami template consists of a double-layer plate (42 nm × 41.2 nm), a 6-helix 90° arc-shaped track (~32 nm in radius), and a 10-helix rotary bundle (45 nm in length). The arc-shaped track is immobilized on the top surface of the plate, whereas one end of the rotary bundle is flexibly linked to the plate through adjacent scaffold crossovers (Supplementary Figs. 1 and 2). The rotary module contains a AuNR (yellow, 10 nm × 38 nm) anchored on the rotary bundle and the arc-shaped track dressed with five rotation footholds (rfhs, rfh1–rfh5). A foot strand (Fr) is extended from the other end of the rotary bundle, 32 nm away from the flexible linker. The five rotation footholds spaced by 12.6 nm on the arc-shaped track define five rotation positions 1–5 (see Fig. 1a and b). Thecorresponding rotation angles for positions 1–5 are 15°, 37.5°, 60°, 82.5°, and 105°. The walking module contains a AuNR (blue, 10 nm × 38 nm) fully decorated with foot strands for walking (Fw) and six rows of walking footholds (wfhs, wfha–wfhf) extended from the bottom surface of the plate. These six foothold rows define five walking positions I–V that are evenly separated by 7 nm (Fig. 1a, b, and Supplementary Fig. 2d). The walker AuNR is assembled on the plate through DNA hybridization between the Fw and two neighboring rows of wfhs. Figure 1c presents the transmission electron microscopy (TEM) image of the DNA origami template structures. Each structure contains three components: bundle, arc, and plate. Figure 1d shows the TEM images of the UNITs, in which the AuNRs are assembled on the individual DNA origami templates. Additional structural information is presented in Supplementary Figs. 1–3.

The reversible rotation and walking mechanisms are displayed in Fig. 1e and f, respectively. Two independent sets of DNA fuel strands are designed to power the stepwise rotation and walking through toehold-mediated strand displacement reactions^8^, respectively (see Supplementary Figs. 4 and 5). The rotation process proceeds in a “release and capture” manner^31^. Blocking (rB1–rB5) and removal (rR1–rR5) strands are utilized for activation and deactivation of rfh1–rfh5, respectively. As shown in Fig. 1e, the rotary bundle is first fixed at position 1 through DNA hybridization between Fr (black) and rfh1 (brown), while all other four rfhs are blocked. For rotation from position 1 to 2, rfh2 is activated and rfh1 is blocked upon addition of removal strands rR2 and blocking strands rB1, respectively. Then, Fr is hybridized with the activated rfh2. The rotary bundle clockwisely rotates about 22.5° and reaches position 2. The walking process carries on through “adhesive rolling”^30^. Blocking (wBa–wBf) and removal (wRa–wRf) strands are utilized to control the accessibility of wfha–wfhf. As shown in Fig. 1f, the walker AuNR first resides at the position I with Fw bound to wfha (red) and wfhb (blue). All other four rows of wfhs are blocked. For walking from position I to II, blocking strands wBa and removal strands wRc is added to block wfha and activate wfhc, respectively. The walker AuNR moves forward to interact with the activated wfhc and subsequently reaches position II.

### Optical characterizations of the UNIT motion

**Fig. 2:**
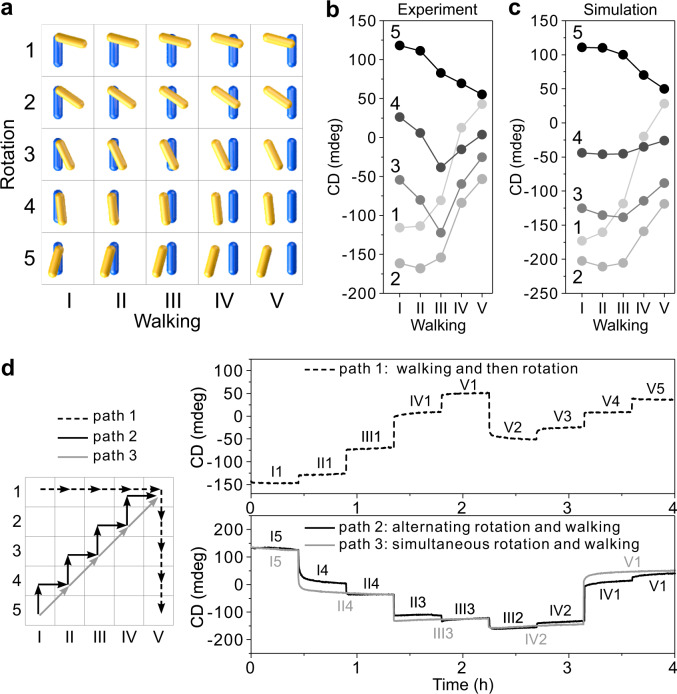
Optical characterizations of the UNITs. **a** 25 states of the UNIT with 5 rotation positions and 5 walking positions. **b** CD intensities extracted at 721 nm at these 25 states. **c** Simulated results for the 25 states. The rotation angles for positions 1–5 are 15°, 35°, 65°, 85°, and 105° in the calculations. **d** Time-course measurements along three paths. Path 1 (black dashed): a walking route followed by a rotation route. Paths 2 and 3: alternating (path 2, black) and simultaneous (path 3, gray) rotation and walking from state I5 to state V1. The arrows indicate the directions of the movements. Concentrations of the AuNRs in all the samples are about 1 nM.

Altogether, five rotation positions and five walking positions establish 25 different states for the UNIT as depicted in Fig. 2a. The configuration changes among different states on the nanoscale give rise to the near-field coupling changes between the two AuNRs within the UNIT, which is a plasmonic chiral object in nature^58,59^. These different states can be optically discriminated against by circular dichroism (CD) spectroscopy^59,64^. The CD spectra (see Supplementary Fig. 6) at the 25 states have been measured using a Jasco-1500 CD spectrometer. Figure 2b shows the experimental CD intensities extracted at 721 nm. Figure 2c presents the theoretical results, which agree qualitatively well with the experimental results. Next, the dynamic transitions among different states are optically monitored. As shown in Fig. 2d, path 1 first follows a walking route and then a rotation route. Starting at state I1, the walker AuNR strides from the left to the right on the origami plate and halts at state V1. Afterward, the rotary AuNR stepwisely rotates from state V1 to state V5. As presented by the dashed black curve in Fig. 2d, the time-course CD measurements reveal nine distinct states in the expected order. Subsequently, the walking and rotary modules that are operated alternatingly (path 2) and simultaneously (path 3) are examined. As shown in Fig. 2d, the time-course CD measurements along these two paths presented by solid black and gray curves exhibit a close resemblance, indicating that the UNIT can carry out the rotation and walking motion in concert with high fidelity.

### Dimerization of the UNITs and motion of the DIMER

**Fig. 3:**
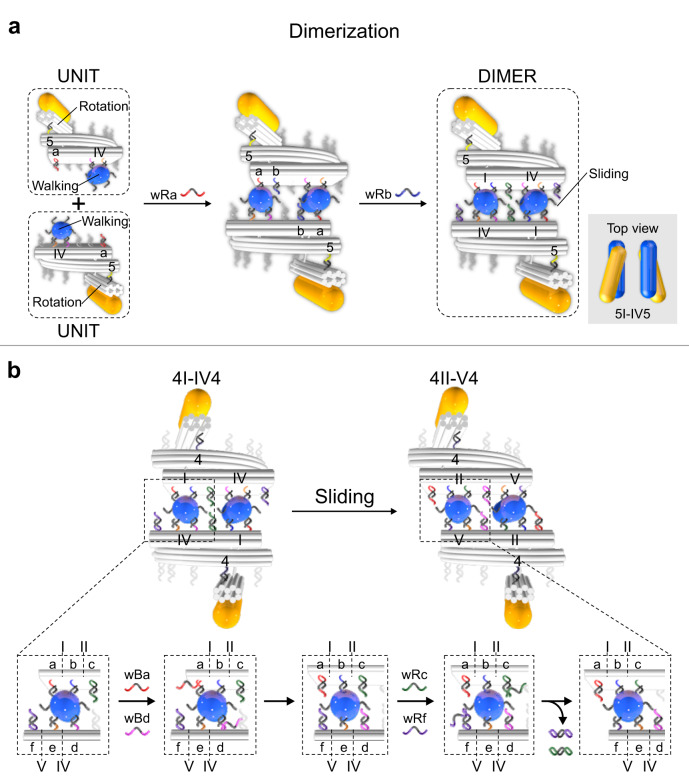
Dimerization of the UNITs. **a** Schematic of the dimerization of the UNITs at state IV5. Wfha in the UNITs is activated through the addition of wRa. The interaction between wfha in one UNIT and the walker AuNR in the other UNIT leads to the crosslinking of the two UNITs in an antiparallel fashion, forming a DIMER. Wfhb is activated by wRb to stabilize the DIMER at state 5I-IV5. The two walking modules are merged into a sliding module. Inset: top view of the AuNRs in the DIMER at state 5I-IV5. **b** Working principle of the coordinated sliding from state 4I-IV4 to 4II-V4.

Next, the dimer interface of the walking module is activated to create a new structure from two UNITs, called DIMER. As shown in Fig. 3a, wfha in the UNIT at state IV5 are activated by wRa. The interaction between wfha and the walker AuNR at position IV within the same UNIT is hindered, as their relative distance is as large as 19.5 nm. In contrast, the cooperative interaction between wfha in one UNIT and the walker AuNR in the other UNIT induces the crosslinking of the two UNITs in an antiparallel fashion, leading to the formation of a DIMER. Subsequently, wRb are added to stabilize the DIMER at state 5I-IV5. To evaluate the relative orientations between the UNITs within the individual DIMERs after crosslinking, gold nanoparticles (AuNPs, 10 nm) are utilized as markers for structural analysis by TEM. The detailed information is shown in Supplementary Figs. 7–9. Through dimerization, a new type of linear motion, which is coordinated sliding between the two walking modules, is conferred at the dimer interface. The working principle of reversible sliding is depicted in Fig. 3b. The two rotary modules in the DIMER can collectively carry out rotations along with two opposite directions with relative angle changes at 45° intervals, irrespective of the motion performed by the sliding module. For instance, as shown in Fig. 3b, starting at state 4I-IV4, the two walker AuNRs are bound in between two origami plates through the same combination of four wfhs, in which wfha and wfhb are from one UNIT, as well as wfhd and wfhe are from the other UNIT. Blocking strands wBa and wBd are added to deactivate wfha and wfhd, respectively. The two walker AuNRs are bound to wfhb and wfhe in an antiparallel manner to maintain the dimeric structure. Then, removal strands wRc and wRf are added to activate wfhc and wfhf, respectively. The Fw on each AuNR capture the newly activated wfhs and the DIMER slides to state 4II-V4. For each sliding step, a 14 nm displacement between the two UNITs is introduced.

### Optical characterizations of the DIMER motion

**Fig. 4:**
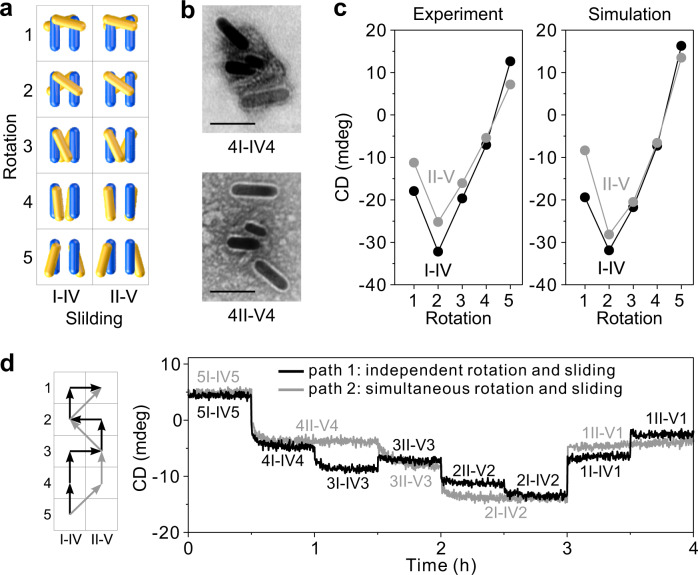
Optical characterizations of the DIMERs. **a** 10 states of the DIMER with 5 rotation positions and 2 sliding positions. **b** Representative TEM images of the DIMERs at states 4I-IV4 and 4II-V4, respectively. Scale bar: 50 nm. **c** Experimental and simulated CD results of the DIMERs extracted at 721 nm at these 10 states. The concentration of the AuNRs in each sample is about 0.2 nM. **d** Time-course measurements of the DIMERs transitioning among different states, following independent (path 1, black) and simultaneous (path 2, gray) rotation and sliding. The concentrations of the AuNRs in the two samples are about 0.1 nM.

Five collective rotation positions and two sliding positions give rise to 10 distinct states for the DIMER as depicted in Fig. 4a. Figure 4b (see also Supplementary Fig. 10) shows the TEM images at the representative states 4I-IV4 and 4II-V4, respectively. Four AuNRs are clearly visible in the individual structures. The configurations of the DIMERs are deformed due to the drying process of the structures on the TEM grids. Figure 4c presents the experimental and theoretical CD intensities extracted at 721 nm at these 10 states, which show a good agreement. The experimental CD spectra are presented in Supplementary Fig. 11. The details of CD calculations and the influence of the parallel dimers on CD can be found in Supplementary Fig. 12. The DIMER can be regulated along different routes through independent (path 1) or simultaneous (path 2) control of the rotary and sliding modules as demonstrated by the time-course measurements in Fig. 4d. Notably, the CD intensities and kinetics at the common states along the two paths show close resemblance. This proves that the DIMER can execute as well-controlled motion as the UNIT does.

### Oligomerization of the UNITs and regulation of the OLIGOMERs

**Fig. 5:**
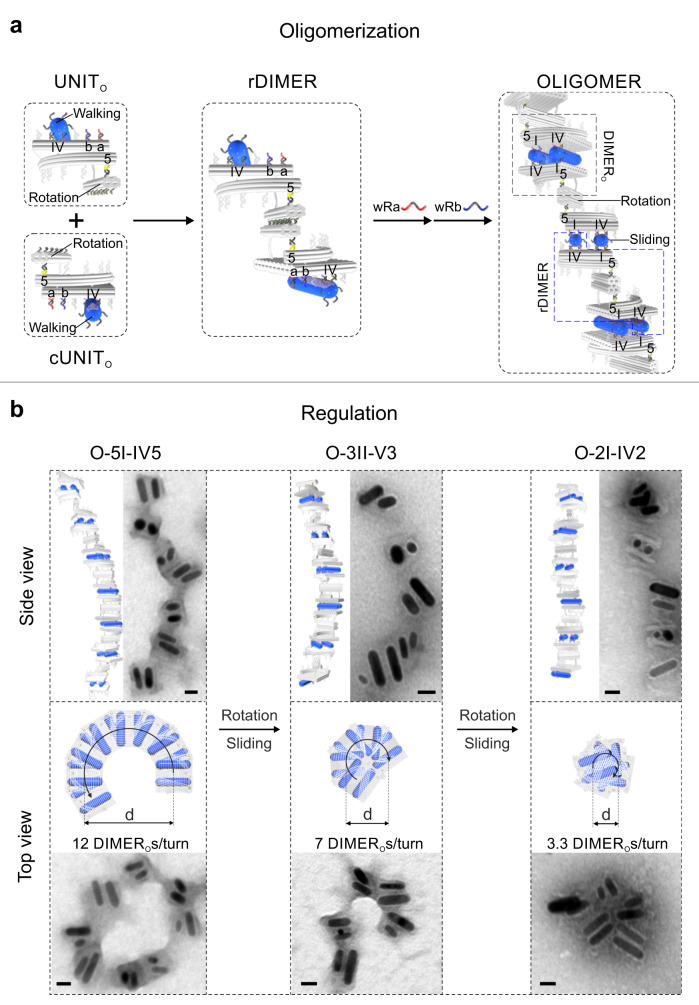
Oligomerization of the UNITs for high-order structures. **a** Schematic of the oligomerization of the UNIT_O_s. The rotary bundles in UNIT_O_ and cUNIT_O_ are functionalized with complementary strands (10 olive strands in UNIT_O_ and 10 dark gray strands in cUNIT_O_). Both UNIT_O_ and cUNIT_O_ are at state IV5. The intermediate dimeric structure is called rDIMER. The final OLIGOMER is at state O-5I-IV5, containing both helical queues of rDIMERs and DIMER_O_s. **b** Regulation of the OLIGOMERs. Schematics and TEM images of the side views and top views of the OLIGOMERs at different states. The OLIGOMERs reconfigure from state O-5I-IV5 to O-3II-V3, and then to state O-2I-IV2 through simultaneous rotation and sliding processes.

To generate higher-order architectures that are named OLIGOMERs, the UNITs are oligomerized by activating the dimer interfaces of both the rotary and walking modules. Here, UNIT and DIMER without the anchored rotary AuNRs are named UNIT_O_ and DIMER_O_, respectively. As shown in Fig. 5a, dimerization of the rotary modules is enabled through the functionalization of complementary DNA strands on the rotary bundles in UNIT_O_ and cUNIT_O_. UNIT_O_ and cUNIT_O_ are mixed in a 1:1 ratio and the intermediate dimeric structure (rDIMER) is depicted in Fig. 5a. The TEM images of the rDIMERs and DIMER_O_s are presented in Supplementary Fig. 13. Together with dimerization of the walking modules, OLIGOMERs are formed, containing helical queues of rDIMERs and helical queues of DIMER_O_s. The OLIGOMERs can reconfigure from state O-5I-IV5 to state O-3II-V3, and then to O-2I-IV2 through simultaneous rotation and sliding processes (see Supplementary Fig. 14). Figure 5b presents the TEM images of the OLIGOMERs at these three states in different views. The side views show linear-like structures in various lengths, resulting from different degrees of oligomerization (see Supplementary Fig. 15). Circular structural profiles in different diameters (d) are observed in the top views. In response to the specific DNA fuels, in an OLIGOMER, the collective motion of the sliding modules results in the relative displacements between the adjacent rDIMERs, while the collective motion of the rotary modules leads to the relative twisting between the adjacent DIMER_O_s. As the rotation angle between two adjacent DIMER_O_s within the OLIGOMER at state O-5I-IV5 is 30°, 12 DIMER_O_s complete one full helical turn (see Supplementary Fig. 14b). From position 5 to 3 and then to 2, the relative twisting leads to the decrease of the number of DIMER_O_s per turn from 12 to 7 and to 3.3 accordingly (Fig. 5b and Supplementary Fig. 14b). As a result, the OLIGOMER shrinks its lateral dimension and becomes more compact. The diameter (d) of the OLIGOMER is also influenced by the sliding modules, as the two sliding positions in each DIMER_O_ correspond to different relative lateral distances between the adjacent rDIMERs (see Supplementary Fig. 14b). Additional schematics and TEM images of the OLIGOMERs in three-dimensional views are shown in Supplementary Fig. 16. Time-course CD measurements of the dynamic transitions among the three states are presented in Supplementary Fig. 17.

## Discussion

In this work, we have demonstrated dimerization and oligomerization of DNA-assembled building blocks for controlled multi-motion in high-order architectures. The monomeric UNIT contains walking and rotary modules. Activation of the walking dimer interface enables the formation of a DIMER from two UNITs and meanwhile introduces the sliding module. Activation of the dimer interfaces of both the walking and rotary modules leads to the formation of OLIGOMERs, which can undergo collective rotation and sliding processes. The collective motion also enforces relative twisting within the structures and leads to varied lateral dimensions. The hierarchical assembly through activation of different dynamic dimer interfaces provides a pathway to achieve complex and controlled multi-motion in high-order structures.

## Methods

### Materials

Single-stranded scaffold DNA (p8064) was purchased from tilibit nanosystems. Staple, blocking, and removal strands were purchased from Sigma-Aldrich. Agarose for electrophoresis and SYBR Gold nucleic acid stain were purchased from Life Technologies. Uranyl formate for negative TEM staining was purchased from Polysciences, Inc.

### Design and assembly of the DNA origami template structures

The UNIT was designed using caDNAno software^65^. It consisted of a 10-helix bundle, a 6-helix arc, and a double-layer plate arranged in a ‘honeycomb’ lattice, which was connected through scaffold crossovers (see Supplementary Fig. 1 and Supplementary Dataset 1). The DNA origami was assembled from 10 nM scaffold strands and 100 nM of each set of the staple strands in a 0.5× TE buffer with 12 mM MgCl_2_ and 5 mM NaCl using a 20 h annealing program (85 °C 5 min, 70 °C–61 °C −1 °C/min, 60 °C–51 °C −1 °C/1 h, 50 °C–22 °C −1 °C/20 min, and held at 15 °C). The DNA origami structures were purified by 0.7% agarose gel electrophoresis in a 0.5× TBE buffer with 11 mM MgCl_2_ for 3 h at 8 V/cm.

### DNA functionalization of the AuNRs and AuNPs, as well as assembly of the UINTs

AuNRs were purchased from Sigma-Aldrich (Cat no. 716812). Functionalization of the AuNRs with thiolated DNA was carried out following a low pH procedure^30^. Basically, thiolated DNA strands were incubated with TCEP [tris(2-carboxyethyl)phosphine] for 2 h. The ratio of DNA: TCEP was 1:200. Second, the AuNRs (1 nM, 1 mL) were spun down and the supernatant was removed. The AuNRs were then mixed with thiolated DNA strands (100 μM, 30 μL). In total, 845 μL modification buffer (0.59× TBE (tris-(hydroxymethyl)-aminomethane, borate, ethylenediaminetetraacetic acid, 0.023% SDS, pH = 3) was added. 10 mL, 5 M NaCl was added every 10 min for nine times. 10 mL, 5 M NaOH was added subsequently to adjust the pH value to ~8, and the final concentration of NaCl reached 0.5 M. The AuNRs functionalized with DNA were then purified by centrifugation. Five times of centrifugations at a rate of 8000×*g* for 30 min were carried out. Each time, the supernatant was carefully removed and the AuNRs were resuspended in a 0.5× TBE buffer containing 0.02% of SDS. The supernatant was then removed. AuNPs were purchased from Sigma-Aldrich (Cat no. 741957). Functionalization of the AuNPs with thiolated DNA was carried out following a well-established procedure^37^. Basically, after reduced with TCEP, thiol-modified DNA and BSPP-modified AuNPs were incubated at a molar ratio of DNA: AuNPs of 300:1 in a 0.5× TBE buffer solution overnight at room temperature. The concentration of NaCl was slowly increased to 500 mM in the subsequent 24 h. The AuNPs functionalized with DNA were then washed using a 0.5× TBE buffer solution in 100 kDa (molecular weight cut-off) centrifuge filters to remove the free DNA strands. DNA-functionalized AuNRs or AuNPs were mixed with the DNA origami structures in a ratio of 10:1. The mixtures were annealed with the procedure of 35 °C 2.5 h, 34 °C–29 °C −1 °C/3 h, 28–26 °C −1 °C/1.5 h, and held at 25 °C. Gel electrophoresis was used to purify the UNITs under the same condition for the purification of the DNA origami template structures.

### Dimerization of the UNITs

250 μM 0.4 μL wfha strands were added in the purified UNITs (1 nM, 100 μL) and the mixture was annealed with the procedure of 30 °C–28 °C −1 °C/6 h, 30–26 °C −1 °C/6 h, and held at 25 °C. Afterward, 250 μM 0.4 μL wfhb strands were added to the OLIGOMERs and the mixture was incubated overnight at room temperature. Gel electrophoresis was used to purify the DIMERs under the same condition for the purification of the DNA origami template structures. The dimerization yield was 68.7% (see Supplementary Fig. 18).

### CD characterizations

The CD spectra were measured using a Jasco-1500 CD spectrometer with a quartz suprasil cuvette (path length, 10 mm). All measurements were carried out at 25 °C. The concentrations of the blocking and removal strands were 250 μM.

For the CD spectral measurements in Fig. 2b, one sample at state III3 was divided into 25 copies. Each copy was 60 μL and the respective DNA fuels were added to drive the individual systems to their designated positions (Supplementary Table 1). To keep the concentration constant, an equal volume of H_2_O was added, if no DNA strands were added. After each addition, the samples were incubated at 25 °C for about 1 h. The final concentrations of the AuNRs in all the systems were about 1 nM.

The time-course measurements for the UNITs were carried out as follows. Two hundred microliter was used for each UNIT sample. The CD signals at 721 nm were monitored using the time-scan acquisition mode with a data pitch of 1 s. The respective blocking and removal strands were added to enable programmed routes (Supplementary Tables 2 and 3). The initial concentrations of the AuNRs were about 1 nM. After the process, the volume of the sample was increased by about 24.4 μL (10.9%).

For the CD spectral measurements in Fig. 4b, one sample at state 5I-IV5 was divided into 10 copies. The same experimental protocol as that for the UNIT was conducted and the respective DNA fuels were added to drive the individual systems (Supplementary Table 4). After each addition, the samples were incubated at 25 °C for about 1 h. The final concentrations of the AuNRs in all the systems were about 0.2 nM.

The time-course measurements for the DIMERs were carried out as follows. Two hundred microliter was used for each DIMER sample. The CD signals at 721 nm were monitored at the same conditions as those of the UNITs. The respective blocking and removal strands were added to enable programmed routes (Supplementary Table 5). The initial concentrations of the AuNRs were about 0.1 nM. After the process, the volume of the sample was increased by about 26.2 μL (11.6%).

### Oligomerization of the UNITs and regulations of the OLIGOMERs

The purified DNA origami templates for UNIT_O_ and cUNIT_O_ were mixed in a ratio of 1:1. The walker AuNRs and the mixture were annealed with the procedure of 35 °C 2.5 h, 34 °C–29 °C −1 °C/3 h, 28–26 °C −1 °C/1.5 h, and held at 25 °C. Gel electrophoresis was used to purify the rDIMERs under the same condition for the DNA origami template structures. 250 μM 0.4 μL wfha strands were added in the purified rDIMERs (0.5 nM, 100 μL) to form the OLIGOMERs and the mixture was annealed with the procedure of 30 °C–28 °C −1 °C/6 h, 30 °C–26 °C −1 °C/6 h, and held at 25 °C. Finally, 250 μM 0.4 μL wfhb strands were added to the OLIGOMERs and the mixture was incubated overnight at room temperature.

For regulation of the OLIGOMERs (Fig. 5b), one sample of OLIGOMERs at state O-5I-IV5 was divided into 3 copies. Each copy was 40 μL and the respective DNA fuels were added to drive the individual systems to their designated states O-3II-V3 and O-2I-IV2 (Supplementary Table 6), respectively. After each addition, the samples were incubated at 25 °C for about 1 h. The final concentrations of AuNRs in all the systems were about 1 nM.

## Supplementary information

Supplementary Information

Supplementary Data 1

## Data Availability

All the data reported in this paper are available from the corresponding author upon request.
