# Supplementary material for: Dimerization and oligomerization of DNA-assembled building blocks for controlled multi-motion in high-order architectures
Source: Nat Commun. 2021 May 28;12:3207. doi: 10.1038/s41467-021-23532-y (PMC8163789; doi:10.1038/s41467-021-23532-y)
Supplement: Supplementary file 1 — Supplementary Information [file 41467_2021_23532_MOESM1_ESM.pdf]

Supplementary Information for

Dimerization and oligomerization of DNA-assembled building blocks for controlled multi-motion in high-order architectures

Ling Xin[1,2], Xiaoyang Duan[2] & Na Liu[1,2]*

[1] 2. Physics Institute, University of Stuttgart, Pfaffenwaldring 57, 70569 Stuttgart, Germany

[2] Max Planck Institute for Solid State Research, Heisenbergstrasse 1, 70569 Stuttgart, Germany

*To whom correspondence should be addressed. E-mail: na.liu@pi2.uni-stuttgart.de

This PDF file includes:

Supplementary Figures 1–18

Supplementary Tables 1–6

Supplementary data file:

Supplementary Data1 (DNA sequences)

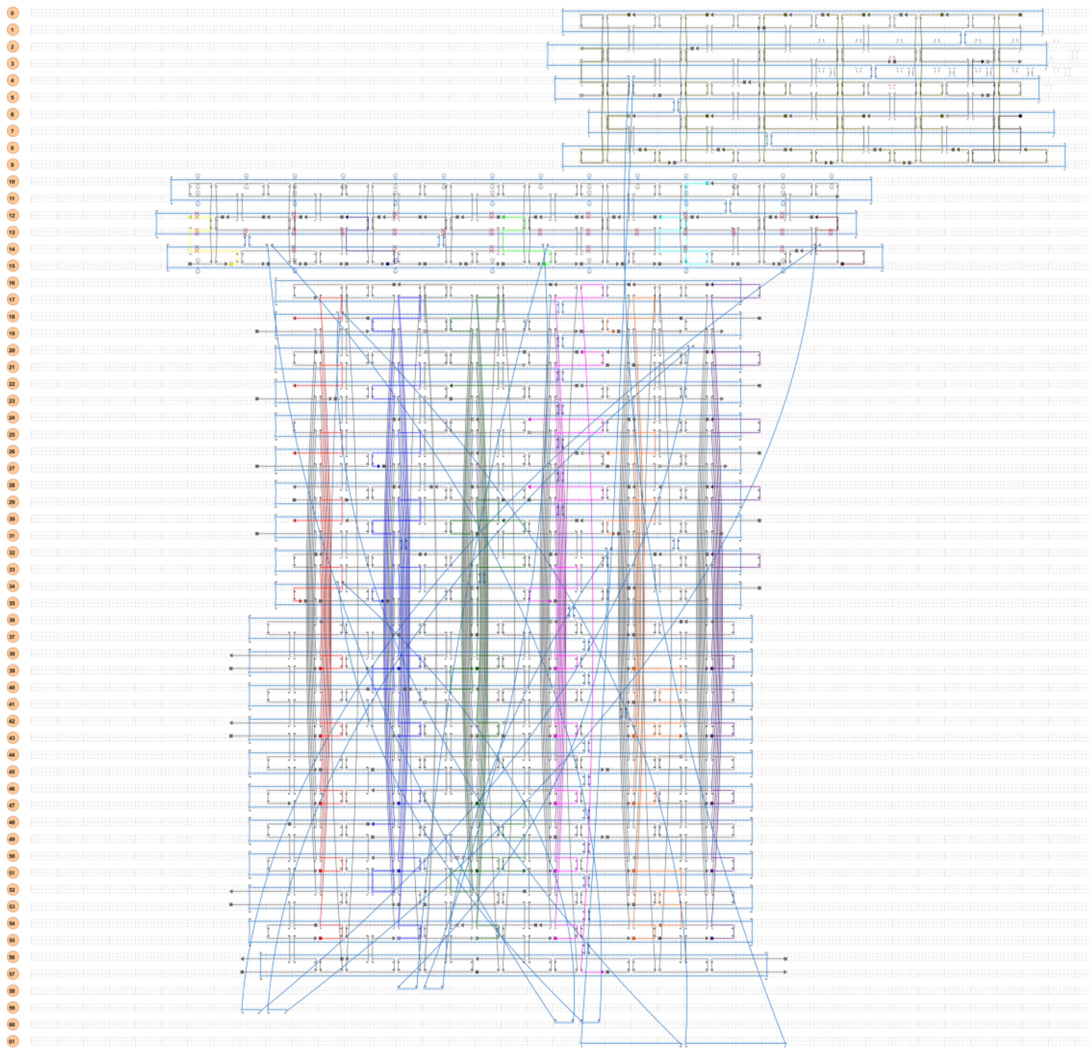

Supplementary Figure 1. Strand routing diagram of the DNA origami template. 12 staples for the capture strands to assemble one AuNR on the rotary bundle (11 of them are in olive). 1 staple for the foot strand for rotation (Fr, black, the 5′ end is modified with a capture strand); 5 staples for the rotation footholds: rfh1 (brown), rfh2 (cyan), rfh3 (green), rfh4 (navy), and rfh5 (yellow). 6 rows of the staples for the walking footholds with five staples in each row: wfha (red), wfhb (blue), wfhc (dark green), wfhd (magenta), wfhe (orange), and wfhf (violet).

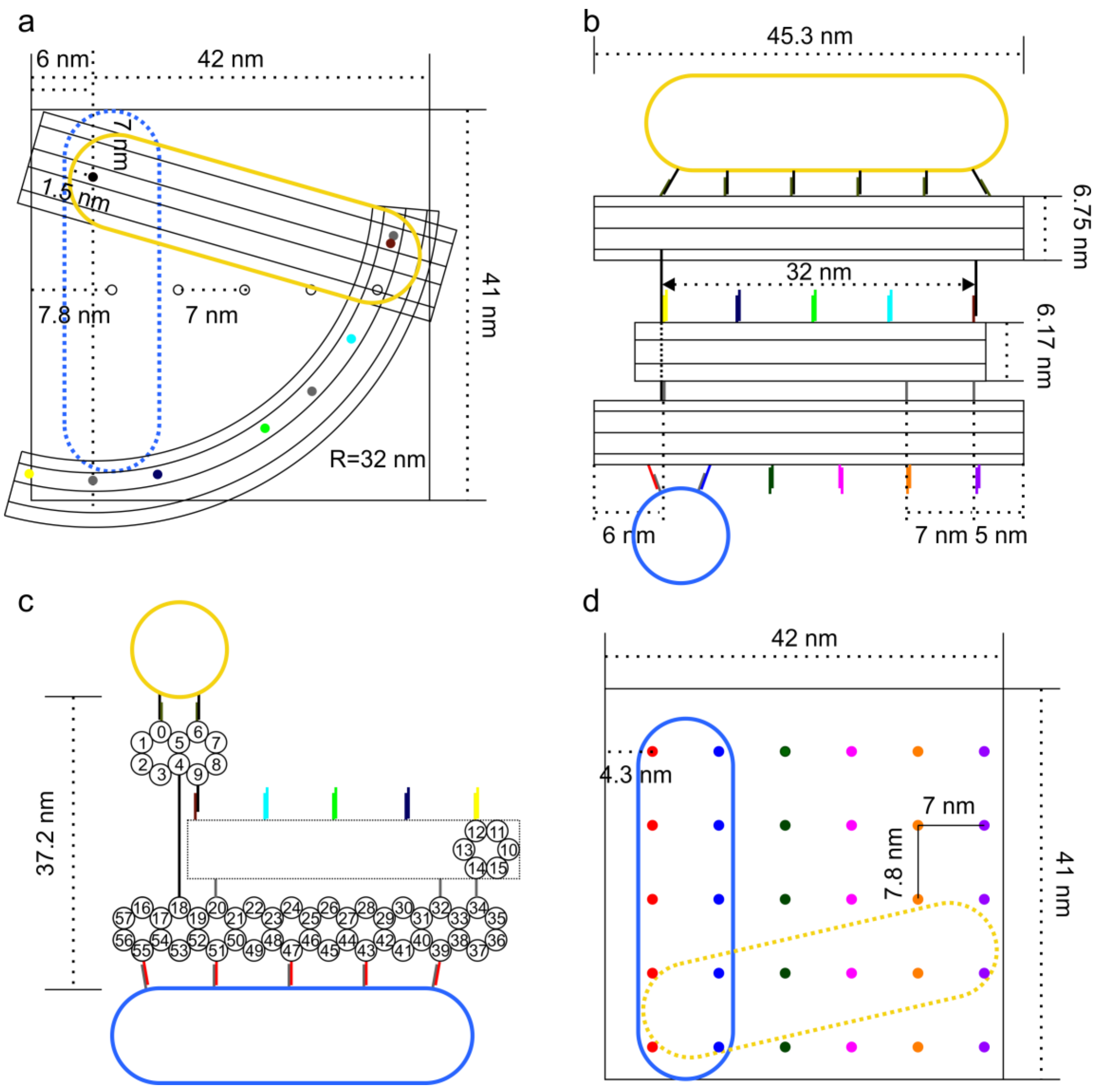

Supplementary Figure 2. Structural details of the UNIT. **a,** Top view. Black dot near the top-left corner represents the linker, which connects the rotary bundle and the plate. Three gray dots represent the linkers, which connect the arc track and the plate. The AuNRs in solid yellow and dash blue represent the rotary AuNR and walker AuNR, respectively. 5 colored dots represent the 5 rfhs to define the rotation positions 1–5 of the rotary AuNR: rfh1 (brown), rfh2 (cyan), rfh3 (green), rfh4 (navy), and rfh5 (yellow). 5 hollow circles show the walking positions I–V of the walker AuNR. The distances for the individual rotation and walking steps are 12.6 nm and 7 nm, respectively. The radius of the arc track is about 32 nm, and the rotation angles of the rotary bundle for positions 1–5 relative to the plate are 15°, 37.5°, 60°, 82.5°, and 105°. **b,** Front view. The rotary AuNR is fixed at position 1 by the interaction between Fr (black) and rfh1 (brown), and all other rfhs are blocked. The lateral distance between the Fr and flexible linker is 32 nm. **c,** Left view. The distance between the surfaces of the two AuNRs is about 37.2 nm. **d,** Bottom view. 6 × 5 dots represent the arrangement of six rows of wfhs and five footholds in each row: wfha (red), wfhb (blue), wfhc (dark green), wfhd (magenta), wfhe (orange), and wfhf (violet).

For TEM imaging, the DNA-assembled structures, UNITs, DIMERs and OLIGOMERs were deposited on freshly glow-discharged carbon/formvar TEM grids. The TEM grids were treated with a uranyl formate solution (2%) for negative staining of the DNA structures. All the structures were imaged using Philips CM 200 TEM operating at 120 kV.

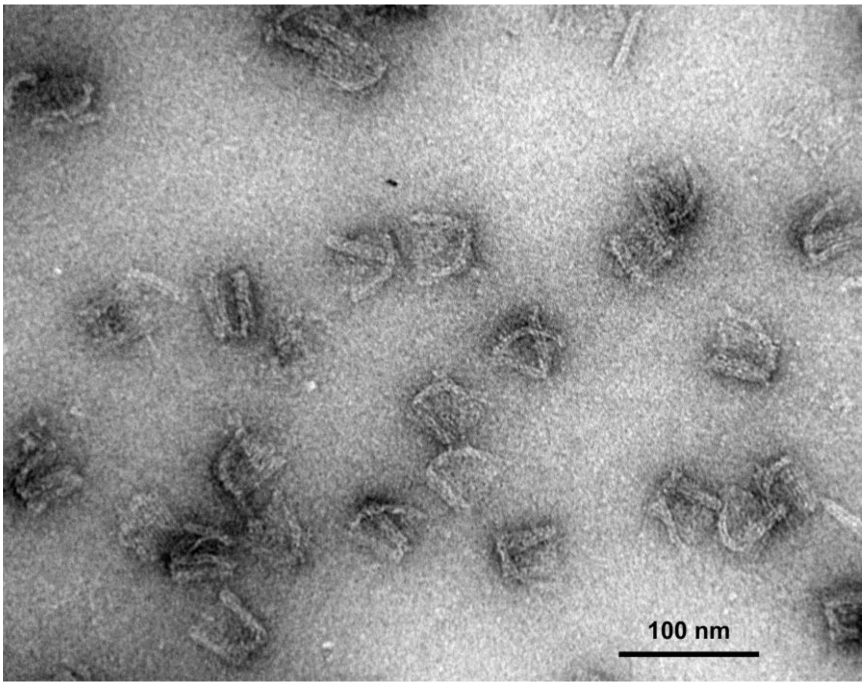

Supplementary Figure 3. TEM image of the DNA origami template structures.

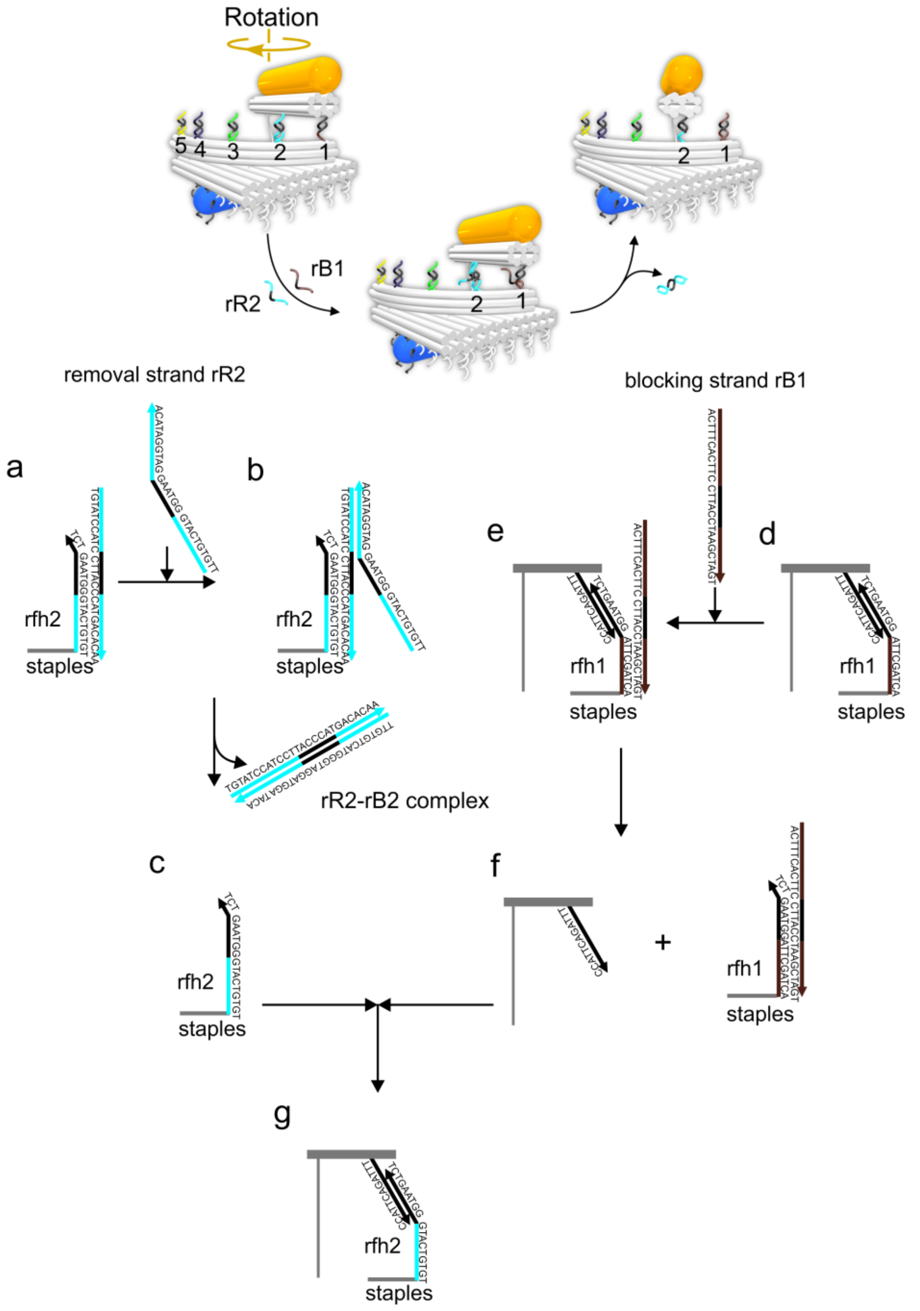

Supplementary Figure 4. Details of the DNA strand displacement reactions for one-step rotation. Each foothold consists of a binding domain (9-nt, black) with an identical sequence and a toehold domain (9-nt, colored). Each blocking strand consists of three domains: an 11 nt-top domain (colored), a 6 nt-middle domain (black), and a 9 nt-bottom domain (colored). The top domain serves as a toehold and the rest two domains function as a blocking segment, which is complementary to the specific foothold for rotation. The corresponding removal and blocking strands are fully complementary to each other.

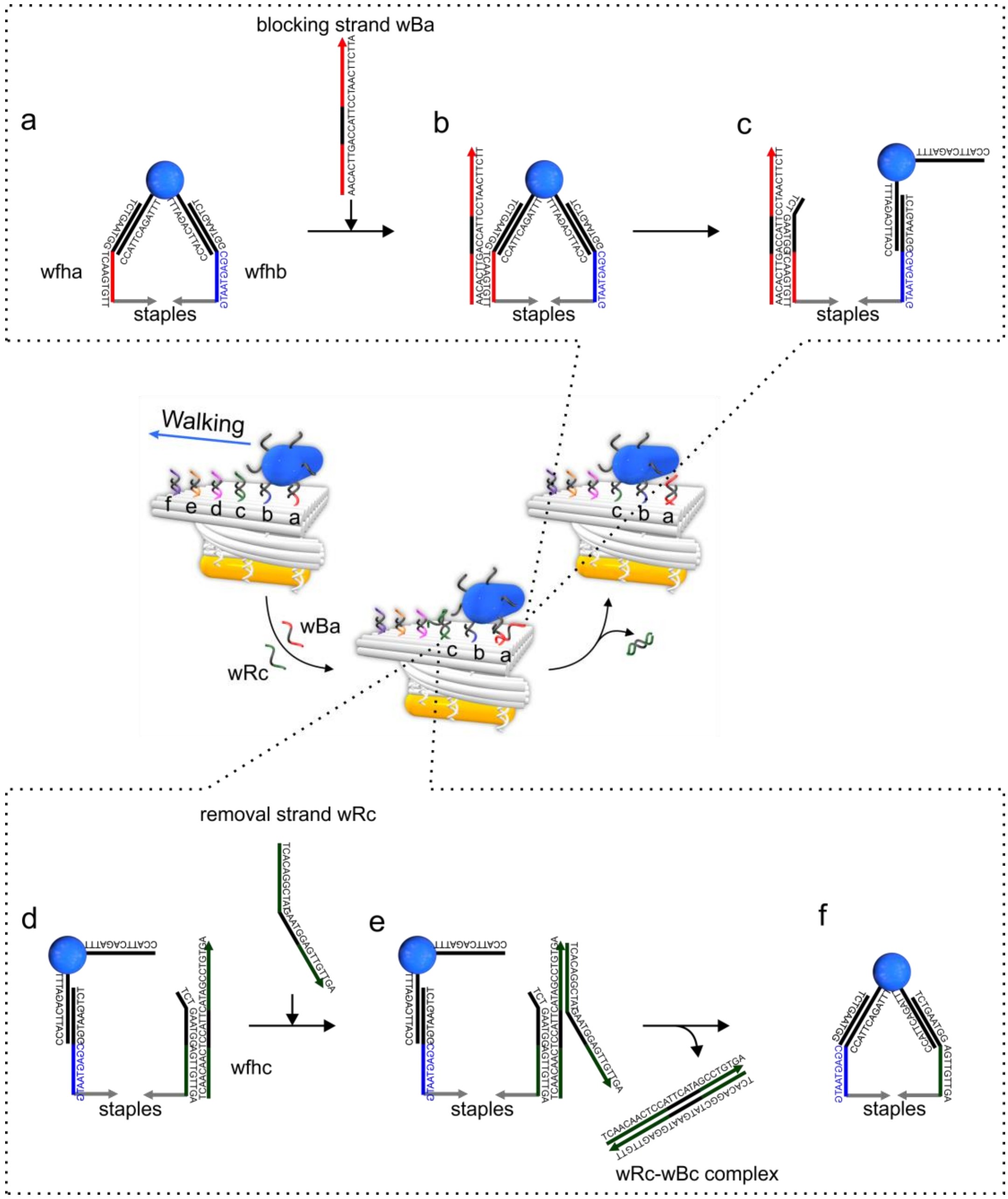

Supplementary Figure 5. Details of the DNA strand displacement reactions for one-step walking. Sequence design for the foot-foothold interactions. The middle and bottom domains of the blocking strands function as a blocking segment, which is complementary to the specific row of footholds for walking.

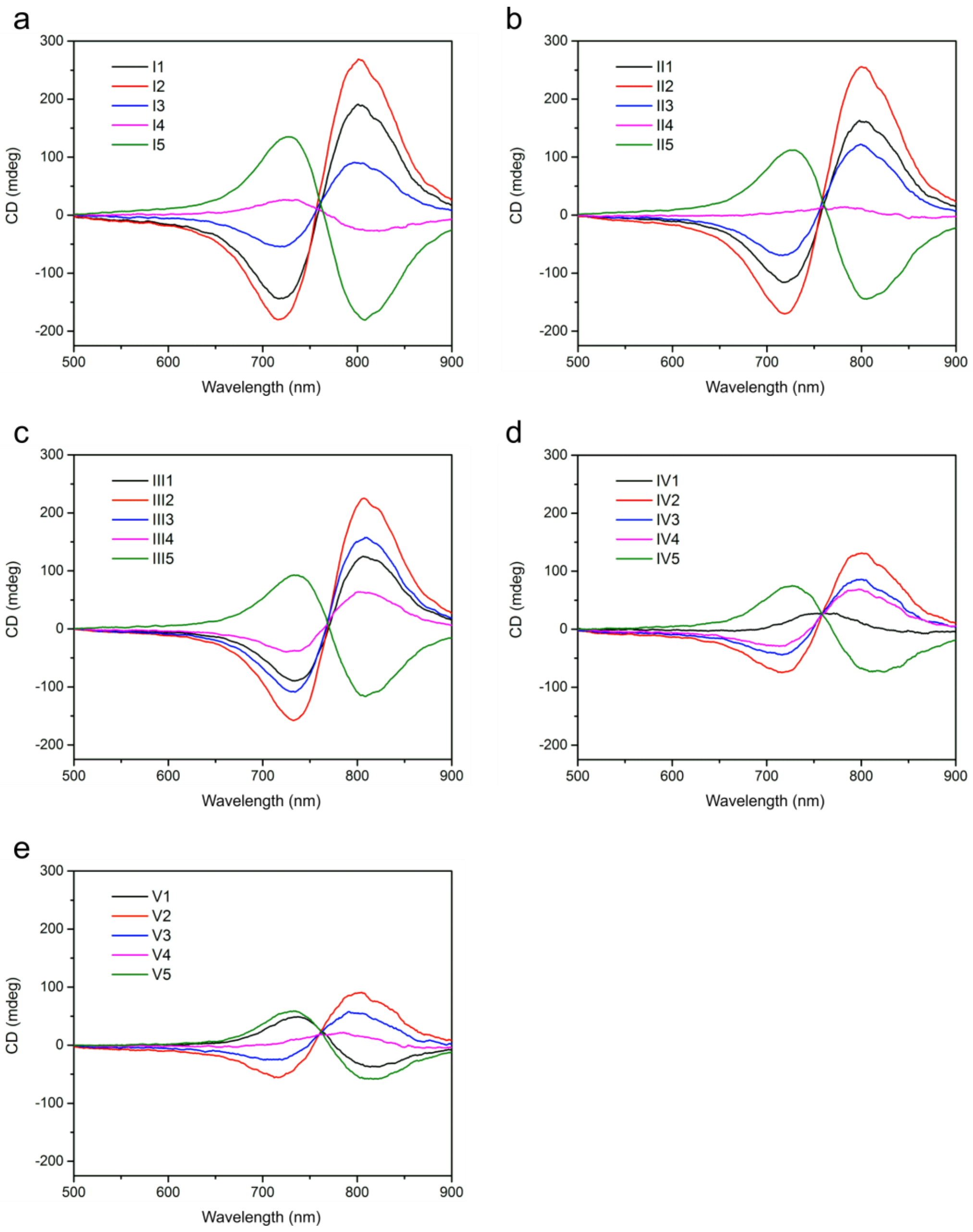

Supplementary Figure 6. CD Spectra of the 25 UNIT samples at 25 states. CD spectra of the samples with the rotary module at 5 different rotation positions and meanwhile the walking module at (**a**) position I, (**b**) position II, (**c**) position III, **(d**) position IV, and (**e**) position V.

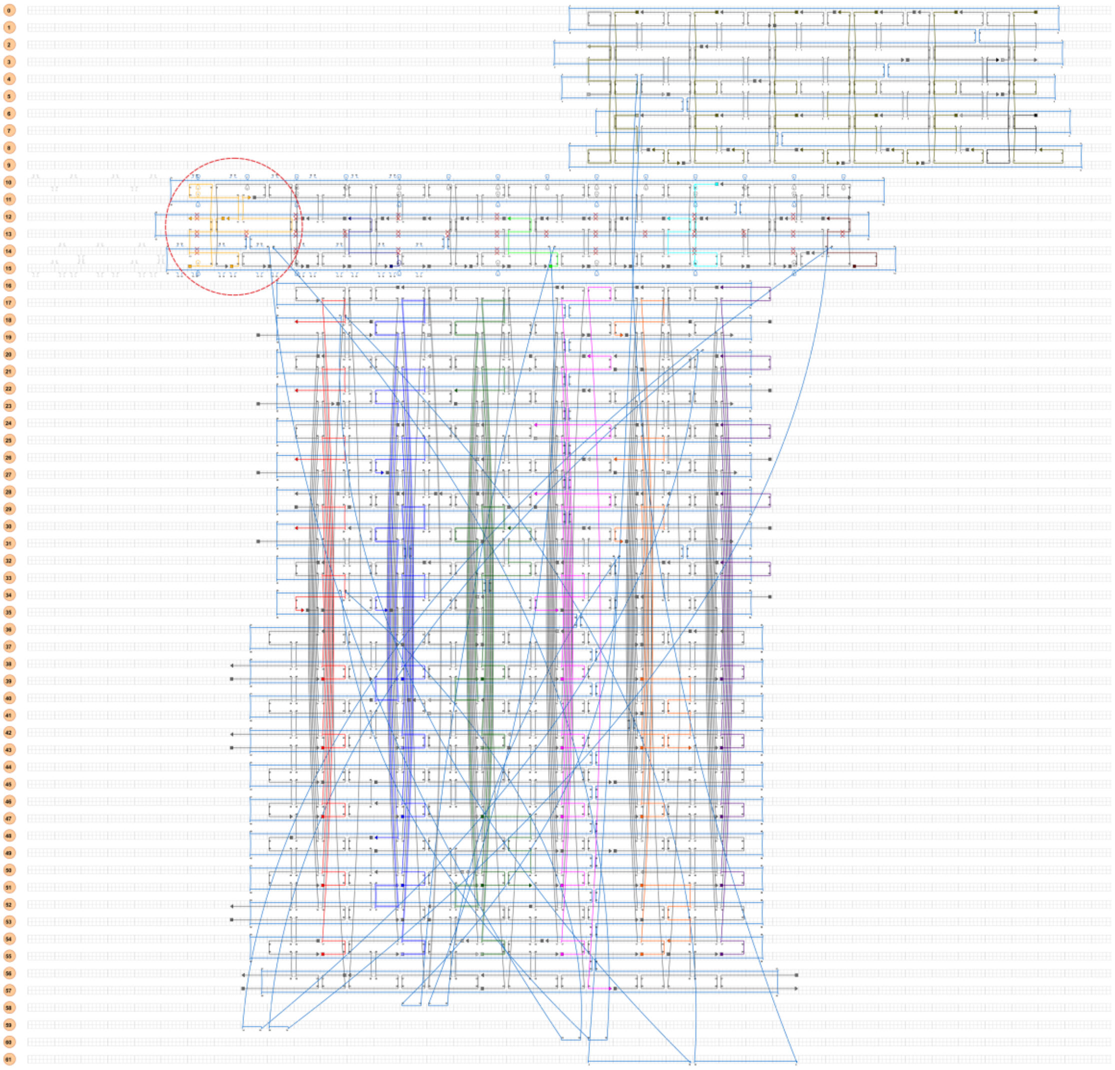

Supplementary Figure 7. Strand routing diagram of the UNIT with a AuNP marker. Three modified staples in dark yellow around position 5 for capturing the AuNP are indicated in the red circle.

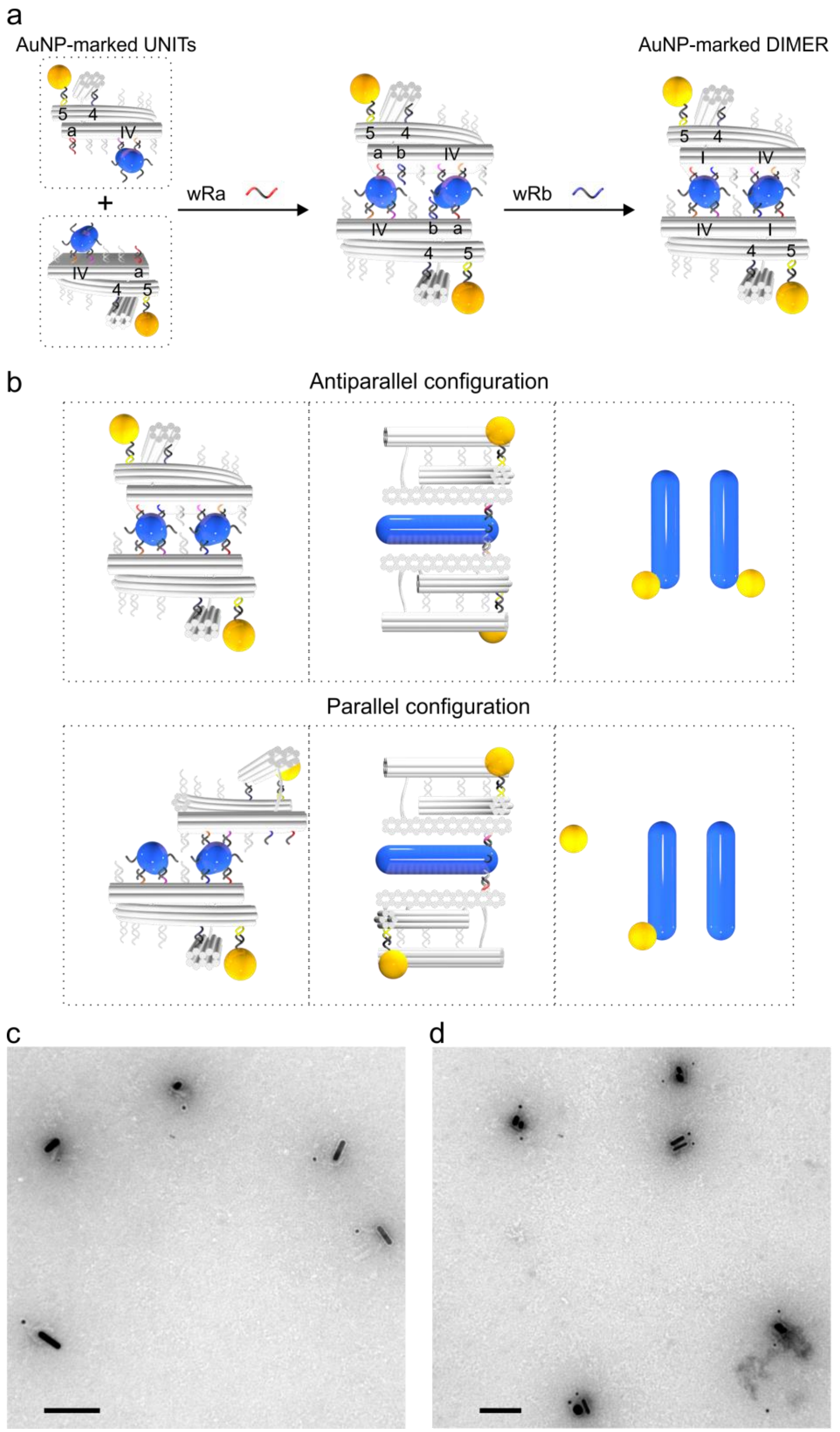

Supplementary Figure 8. AuNP-marked UNITs and DIMER. **a,** Schematic of the assembly of the AuNP-marked DIMER. **b,** Front view, side view, and top view of the dimerized structures in antiparallel and parallel configurations. TEM images of the AuNP-marked UNITs (**c**) and AuNP-marked DIMERs (**d**). Scale bar: 100 nm.

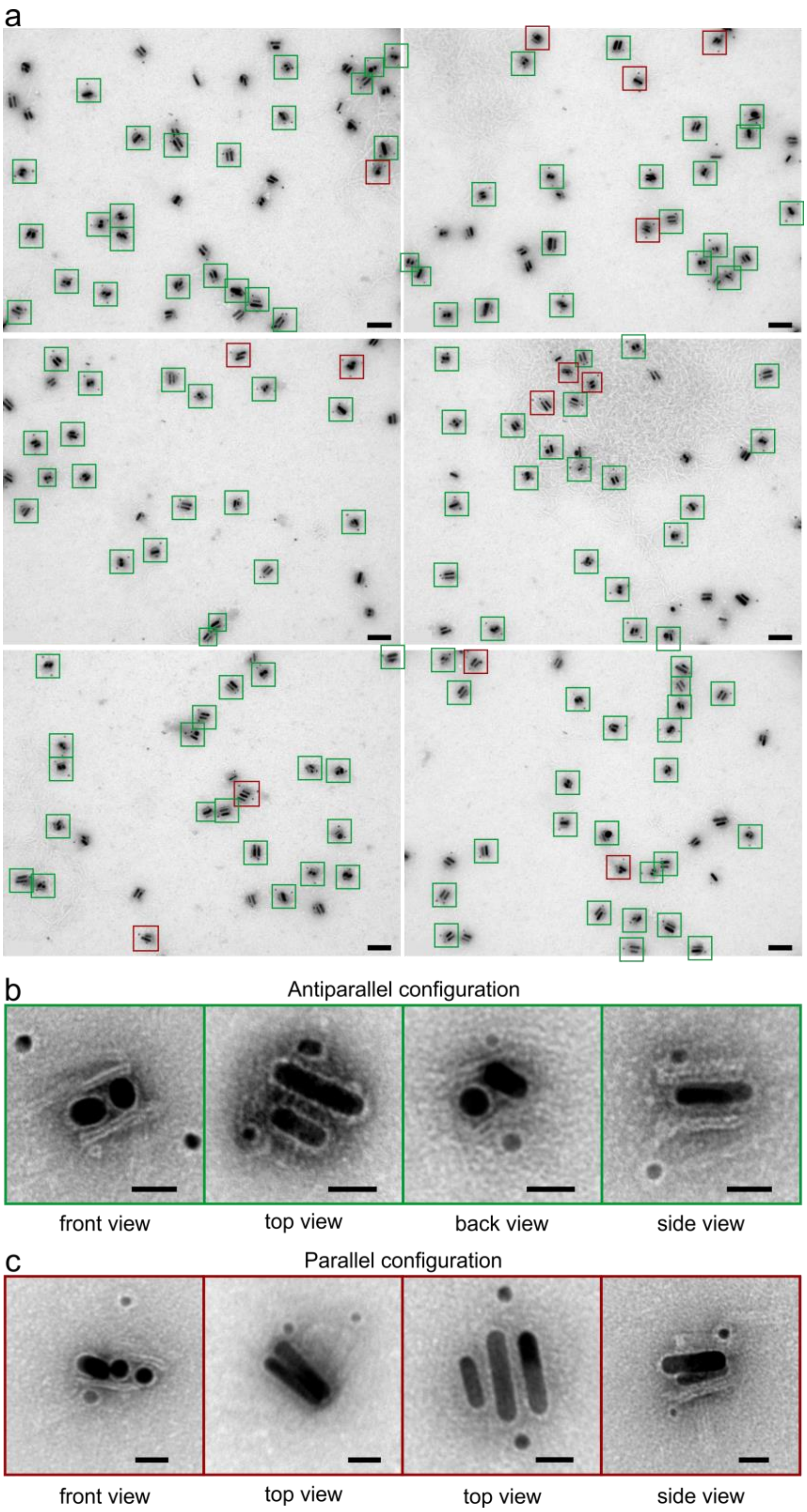

Supplementary Figure 9. TEM images of the AuNP-marked DIMERs. **a**, TEM images of the dimerized structures. Scale bar: 100 nm. Green and red squares highlight the dimerized structures in antiparallel and parallel configurations, respectively. The percentage of the antiparallel dimers is 90.3% through analyzing 144 structures. Enlarged views of the representative dimerized structures in (**b**) antiparallel and (**c**) parallel configurations. Scale bar: 20 nm.

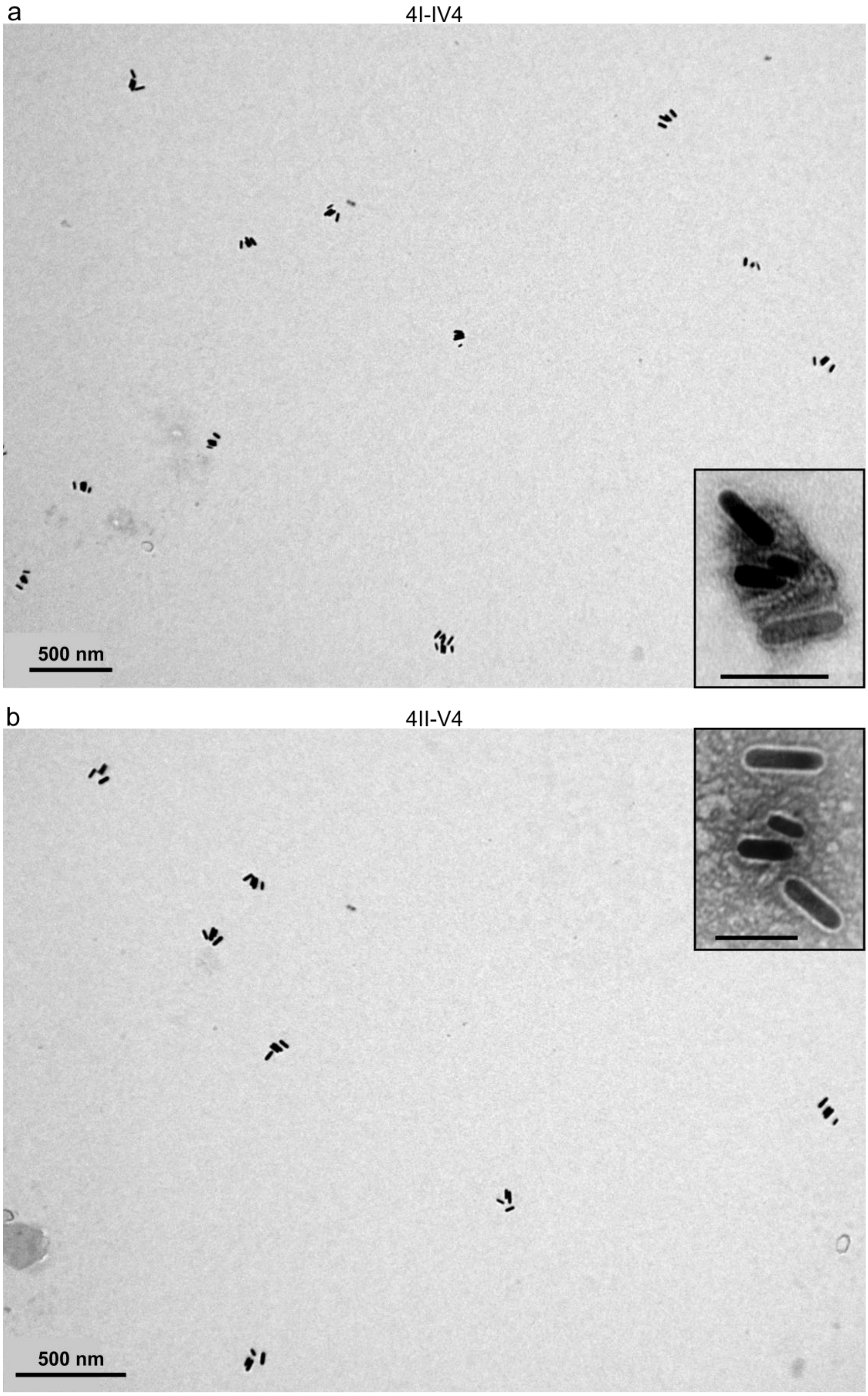

Supplementary Figure 10. TEM images of the DIMERs at states 4I-IV4 (**a**) and 4II-V4 (**b**). Insets: enlarged views of the representative structures at the two states. Scale bar: 50 nm.

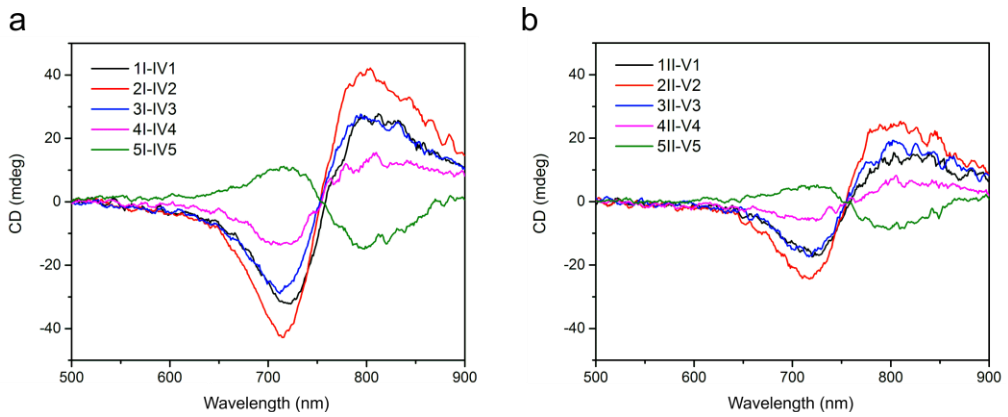

Supplementary Figure 11. CD Spectra of 10 DIMER samples at 10 states. CD spectra of 5 samples with rotary modules at 5 different rotation positions, and the sliding module (**a**) at position I-IV and (**b**) at position II-V.

Theoretical calculations were performed using commercial software COMSOL Multiphysics based on a finite element method. The CD signal was calculated as a difference in extinction for the left- and right-circularly polarized light. Since the plasmonic assemblies were dispersed in solution, we carried out orientational averaging. Averaging over all possible orientations at defined light incidence is equivalent to averaging over all incident directions of light for a nanostructure with defined orientation. It has been demonstrated both analytically and numerically that averaging over six orthogonal directions of light incidence is sufficient to give accurate CD. To account for the inhomogeneous broadening arising from the polydispersity of the AuNRs, the experimental dielectric function of Au was modified by including an additional term:

$$\varepsilon_{effective}(\omega) = \varepsilon_{bulk}(\omega)+\varepsilon_{correction}(\omega) \qquad (1)$$

where the dielectric function of bulk Au, ε_{bulk} is from Johnson and Christy values, and the correction term is introduced following a standard approach:

$$\varepsilon_{correction}(\omega)= \frac{\omega_p^2}{\omega^2+i\omega\gamma} - \frac{\omega_p^2}{\omega^2+i\omega\Gamma_{broad}} \qquad (2)$$

where ω_p=8.754 eV and γ=0.0724 eV are the Drude parameters, respectively. Γ_{broad} is 0.29 eV.

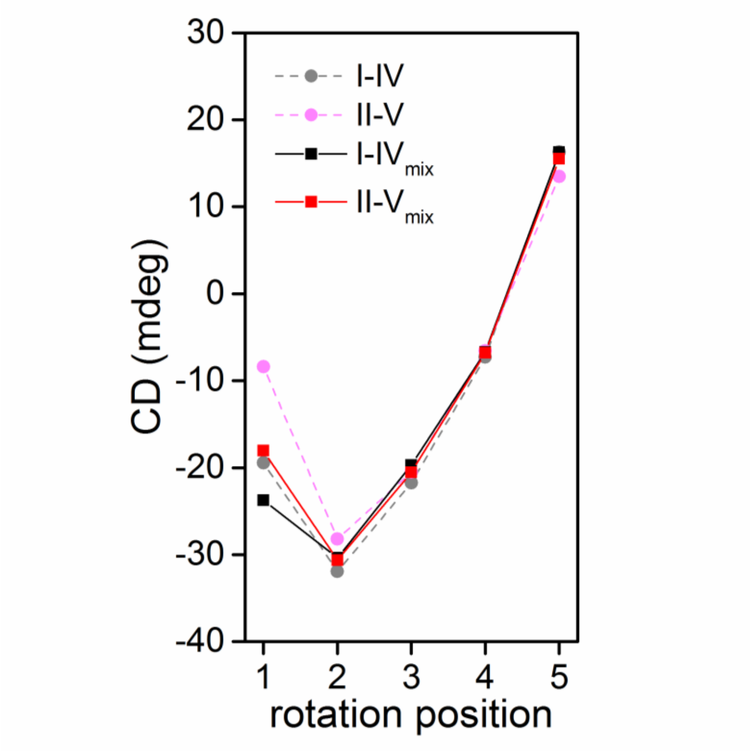

Supplementary Fig. 12. Influence of the parallel dimers on CD at 10 different states. Overall, the presence of the parallel dimers (9.7%, black and red curves) does not have a substantial influence on CD, when compared to the system consisting of pure antiparallel dimers (grey and pink curves).

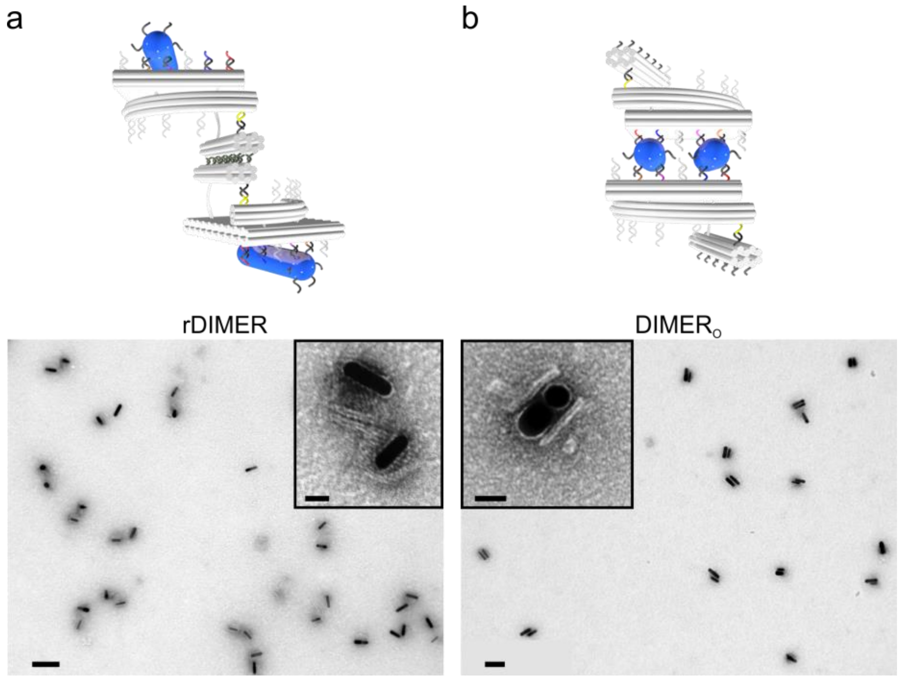

Supplementary Figure 13. rDIMER and DIMER$_O$. Schematics and TEM images of (**a**) rDIMER at state IV5-5IV and (**b**) DIMER$_O$ at state 5I-IV5. rDIMERs are dimeric structures assembled using UNIT$_O$s and cUNIT$_O$s. Scale bar: 100 nm. Insets: enlarged views of the representative structures. Scale bar: 20 nm.

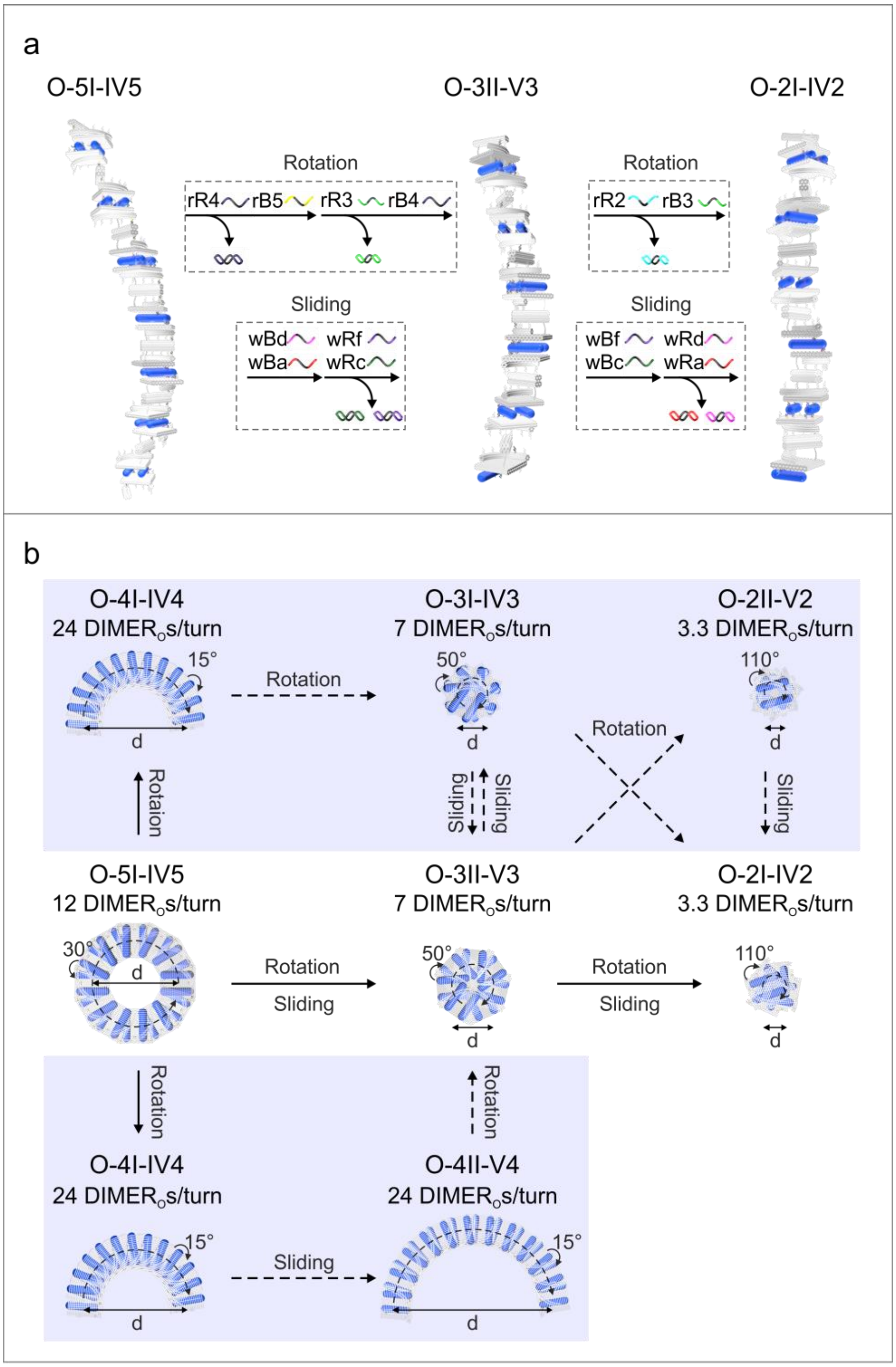

Supplementary Figure 14. Regulation of the OLIGOMERs. **a,** Side views of regulation of an OLIGOMER from state O-5I-IV5 to state O-3II-V3, and then to state O-2I-IV2 through collective rotations and collective sliding powered by strand displacement reactions. **b,** Top view of an OLIGOMER at state O-5I-IV5, which reconfigures to state O-3II-V3, and then to state O-2I-IV2. All states are presented in circular structural profiles with different diameters (d), and the intermediate states are shown in the light blue rectangles. At state O-5I-IV5, the OLIGOMER is a left-handed helical structure. As the rotary angle of position 5 is 105°, the angle between the rotary module and the sliding module is 15° and the angle between two DIMERos is 30°, resulting in 12 DIMERos per turn. For rotation from position 5 to 4, the chirality of the helical structure is

changed to right-handedness, and the number of $DIMER_{OS}$ per turn increases to 24, as the angle between two $DIMER_{OS}$ changes to 15°. Thus, 12 $DIMER_{OS}$ can only form a half turn. For rotation from position 4 to 3, the chirality of the helical structure remains right-handed but the diameter decreases, as the $DIMER_{OS}$ within the helical OLIGOMER folds in by the rotary modules. The number of $DIMER_{OS}$ per turn decreases to 7, as the angle between two $DIMER_{OS}$ changes to 50°. For rotation from position 3 to 2, the angle between two $DIMER_{OS}$ gets larger to 110°, resulting in the number of $DIMER_{OS}$ per turn changes to 3.3. The rotary modules rotate to the middle of the tracks with positions near the diagonals of the $UNIT_{OS}$, folding the structure to be linear-like. From position 5 to 2, the overlap between two $DIMER_{OS}$ is getting larger. Meanwhile, no matter it is sliding from state O-4I-IV4 to O-4II-V4 or from state O-3I-IV3 to O-3II-V3, the number of $DIMER_{OS}$ per turn and angles between the $DIMER_{OS}$ are unchanged, but the distance between adjacent rDIMERs is increased by 14 nm. This enlarges the diameter of the OLIGOMER. When sliding back from state O-2II-V2 to O-2I-IV2, the diameter of the helical structure gets a little bit larger.

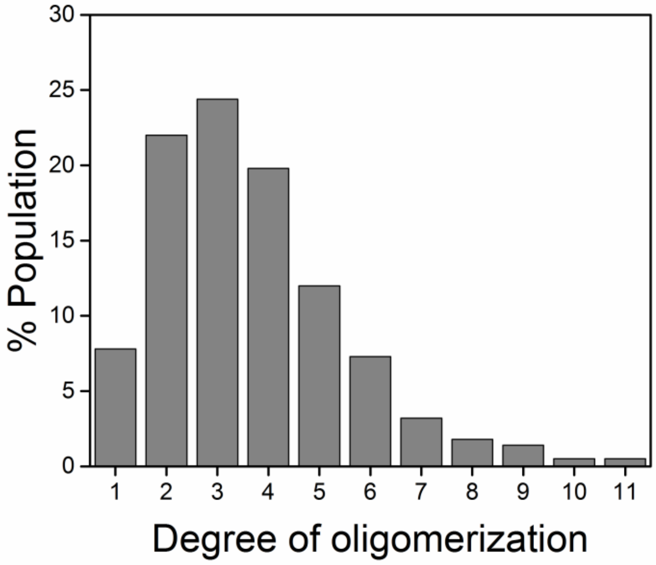

Supplementary Figure 15. Degree of oligomerization evaluated from 217 OLIGOMERs by TEM analysis.

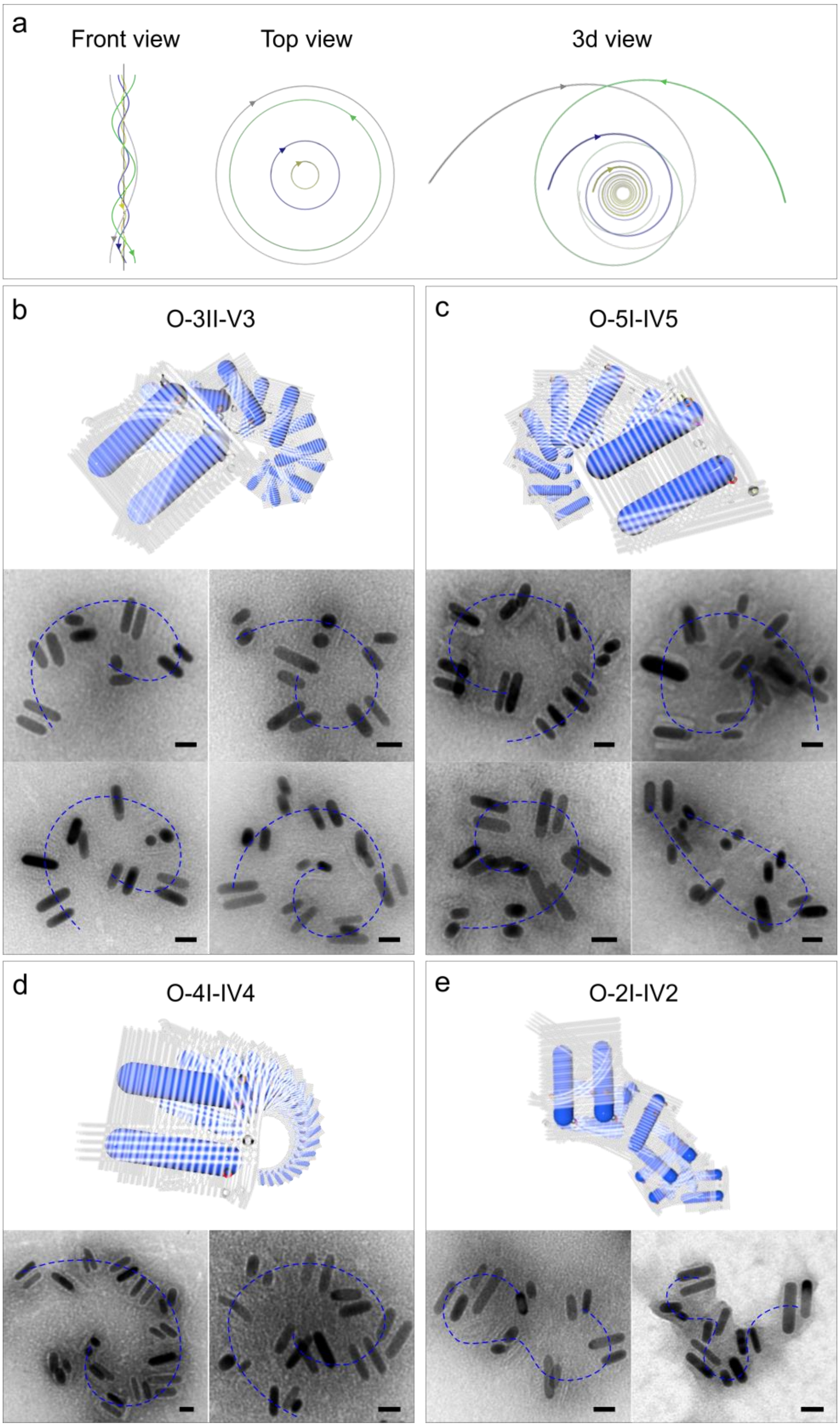

Supplementary Figure 16. 3d views of the OLIGOMERs. **a**, Three different views of the helices in different handedness and diameters with OLIGOMERs at state O-5I-IV5 (green), state O-4I-IV4 (grey), state O-3II-V3 (blue) and state 2I-IV2 (yellow). Schematics and TEM images of OLIGOMERs at (**b**) state O-3II-V3, (**c**) state O-5I-IV5, (**d**) state O-4I-IV4 and (**e**) state O-2I-IV2. The structures in 3D views exhibit spiral configurations (see blue dash lines). Scale bar: 20 nm.

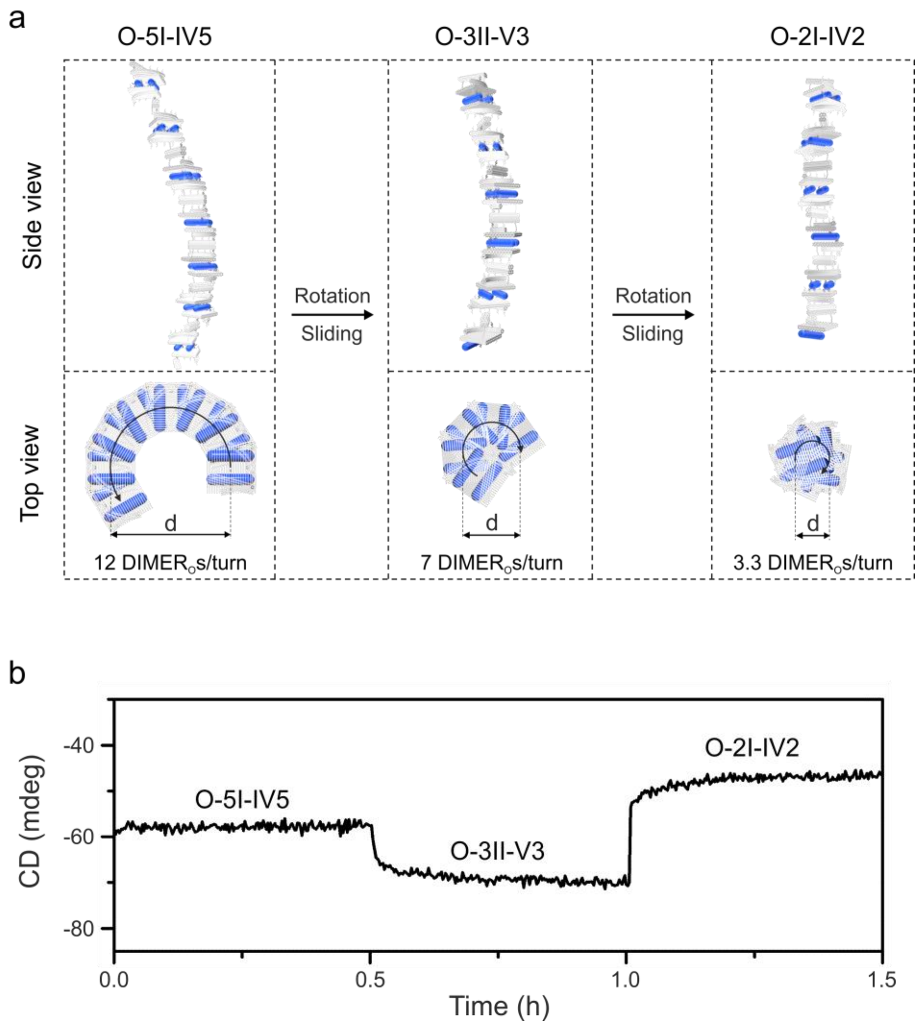

Supplementary Figure 17. Optical characterizations of the collective motion of the OLIGOMERs. **a,** Schematics of the OLIGOMERs at 3 different states. **b,** Time-course measurements of the OLIGOMERs reconfiguring from state O-5I-IV5 to state O-3II-V3, and then to state O-2I-IV2. The CD signal at 750 nm is monitored using the time-scan acquisition mode with a data pitch of 1 s. The sample volume is 97.6 μL. The respective blocking and removal strands are added to enable programmed routes (see Supplementary Table 6). The initial concentration of the AuNRs is about 1 nM.

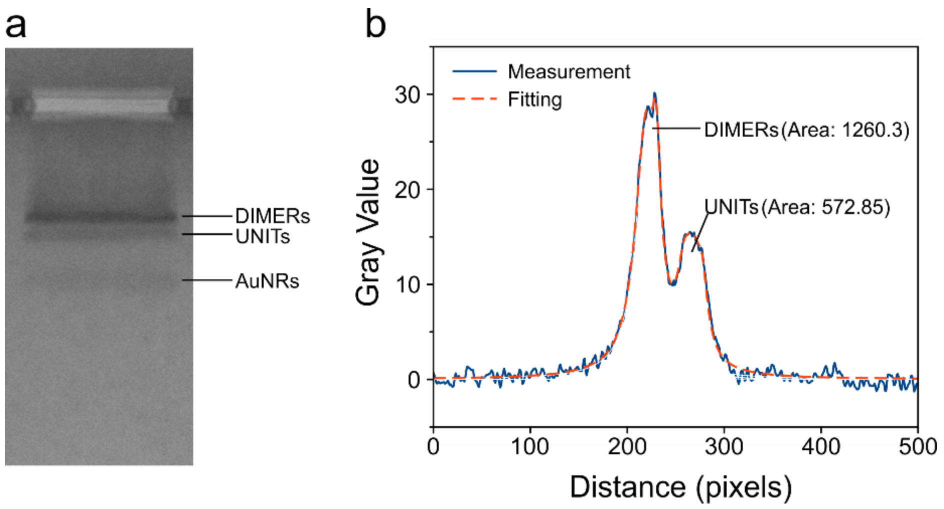

Supplementary Figure 18. Calculation of the dimerization yield. **a**, Agarose gel electrophoresis for characterization of the assembly of the DIMERs. **b**, Gel analysis using the ImageJ and Magicplot (©2016, Magicplot Systems, LLC) software. Analysis is done by plotting the relative densities of the gel bands. The two prominent peaks correspond to the DIMER and UNIT bands in the gel. The dimerization yield is determined by fitting the two peaks and calculating their integrals.

Supplementary Table 1. Additions of DNA fuels for 25 configurations. All the fuel strands have a concentration of 250 μM. The concentrations are the same in Supplementary Tables 3 to 7.

States (III3→ I1)	removal	blocking
III3→ II3	2.0 μL r2	2.0 μL b4
II3→ II2	0.4 μL R2	0.4 μL B3
II2→ I2	2.0 μL r1	2.0 μL b3
I2→ I1	0.4 μL R1	0.8 μL B2
States (III3→ I5)	removal	blocking
III3→ III4	0.4 μL R4	0.4 μL B3
III4→ II4	2.0 μL r2	2.0 μL b4
II4→ II5	0.4 μL R5	0.8 μL B4
II5→ I5	2.0 μL r1	2.0 μL b3
States (III3→ V1)	removal	blocking
III3→ III2	0.4 μL R2	0.4 μL B3
III2→ IV2	2.0 μL r5	2.0 μL b3
IV2→ IV1	0.4 μL R1	0.8 μL B2
IV1→ V1	2.0 μL r6	2.0 μL b4
States (III3→ V5)	removal	blocking
III3→ IV3	2.0 μL r5	2.0 μL b3
IV3→ IV-4	0.4 μL R4	0.4 μL B3
IV4→ V4	2.0 μL r6	2.0 μL b4
V4→ V5	0.4 μL R5	0.8 μL B4
States (III3→ II1)	removal	blocking
III3→ III2	0.4 μL R2	0.4 μL B3
III2→ III1	0.4 μL R1	0.8 μL B2
III1→ II1	2.0 μL r2	2.0 μL b4
States (III3→ I4)	removal	blocking
III3→ II3	2.0 μL r2	2.0 μL b4
II3→ I3	2.0 μL r1	2.0 μL b3
I3→ I4	0.4 μL R4	0.4 μL B3
States (III3→ IV5)	removal	blocking
III3→ III4	0.4 μL R4	0.4 μL B3
III4→ IV5	0.4 μL R5	0.8 μL B4
III5→ IV5	2.0 μL r5	2.0 μL b3
States (III3→ V2)	removal	blocking
III3→ IV3	2.0 μL r5	2.0 μL b3
IV3→ V-3	2.0 μL r6	2.0 μL b4
V3→ V2	0.4 μL R2	0.4 μL B3

Supplementary Table 2. Additions of DNA fuels for reconfiguration of the UNITs (path 1).

Path 1 walking route		
States	removal	blocking
I1→ II1	2.0 μL r3	2.0 μL b1
II1→ III1	2.0 μL r4	2.0 μL b2
III1→ IV1	2.0 μL r5	4.0 μL b3
IV1→ V1	2.0 μL r6	4.0 μL b4
Path 1 rotation route		
States	States	States
V1→ V2	0.4 μL R2	0.4 μL B1
V3→ V3	0.4 μL R3	0.8 μL B2
V3→ V4	0.4 μL R4	0.8 μL B3
V4→ V5	0.4 μL R5	0.8 μL B4

Supplementary Table 3. Additions of DNA fuels for alternating and simultaneous motion of the two modules in the UNIT (path 2 and path 3).

Path 2		
States	removal	blocking
I5→ I4	0.4 μL R4	0.4 μL B5
I4→ II4	2.0 μL r3	2.0 μL b1
II4→ II3	0.4 μL R3	0.8 μL B4
II3→ III3	2.0 μL r4	2.0 μL b2
III3→ III2	0.4 μL R2	0.8 μL B3
III2→ IV2	2.0 μL r5	4.0 μL b3
IV2→ IV1	0.4 μL R1	0.8 μL B2
IV1→ V1	2.0 μL r6	4.0 μL b4

Path 3		
States	removal	blocking
I5→ II4	0.4 μL R4<br>2.0 μL r3	0.4 μL B5<br>2.0 μL b1
II4→ III3	0.4 μL R3<br>2.0 μL r4	0.8 μL B4<br>2.0 μL b2
III3→ IV2	0.4 μL R2<br>2.0 μL r5	0.8 μL B3<br>4.0 μL b3
IV2→ V1	0.4 μL R1<br>2.0 μL r6	0.8 μL B2<br>4.0 μL b4

Supplementary Table 4. Additions of DNA fuels for 10 configurations of the DIMERs.

States	removal	blocking
5I-IV5→ 4I-IV4	0.4 μL R4	0.4 μL B5
4I-IV4→ 3I-IV3	0.4 μL R3	0.8 μL B4
3I-IV3→ 2I-IV2	0.4 μL R2	0.8 μL B3
2I-IV2→ 1I-IV1	0.4 μL R1	0.8 μL B2
States	removal	blocking
5I-IV5→ 5II-V5	2 μL r6, 2 μL r3	2 μL b4, 2 μL b1
5I-IV5→ 4I-IV4	0.4 μL R4	0.4 μL B5
4I-IV4→ 3I-IV3	0.4 μL R3	0.8 μL B4
3I-IV3→ 2I-IV2	0.4 μL R2	0.8 μL B3
2I-IV2→ 1I-IV1	0.4 μL R1	0.8 μL B2

Supplementary Table 5. Additions of DNA fuels for reconfiguration of the DIMERs (path 1 and path 2).

Path 1		
States	removal	blocking
5I-IV5→ 4I-IV4	0.2 μL R4	0.2 μL B5
4I-IV4→ 3I-IV3	0.2 μL R3	0.4 μL B4
3I-IV3→ 3II-V3	1 μL r6, 1 μL r3	1 μL b4, 1 μL b1
3II-V3→ 2II-V2	0.2 μL R2	0.4 μL B3
2II-V2→ 2I-IV2	2 μL r1, 2 μL r4	2 μL b3, 2 μL b6
2I-IV2→ 1I-IV1	0.2 μL R1	0.4 μL B2
1I-IV1→ 1II-V1	3 μL r6, 3 μL r3	3 μL b4, 3 μL b1
Path 2		
States	removal	blocking
5I-IV5→ 4II-V4	0.2 μL R4,<br>1 μL r6, 1 μL r3	0.2 μL B5,<br>1 μL b4, 1 μL b1
4II-V4→ 3II-V3	0.2 μL R3	0.4 μL B4
3II-V3→ 2I-IV2	0.2 μL R2,<br>2 μL r1, 2 μL r4	0.4 μL B3,<br>2 μL b3, 2 μL b6
2I-IV2→ 1II-V1	0.2 μL R1,<br>3 μL r6, 3 μL r3	0.4 μL B2,<br>3 μL b4, 3 μL b1

Supplementary Table 6. Additions of DNA fuels for reconfiguration of the OLIGOMERs.

States	removal	blocking
O-5I-IV5→O-4I-IV4<br>O-4I-IV4→O-3II-V3	0.4 μL R4	0.4 μL B5
	0.4 μL R3<br>2 μL r6, 2 μL r3	0.8 μL B4<br>2 μL b4, 2 μL b1
O-3II-V3→O-2I-IV2	0.4 μL R2<br>4 μL r1, 4 μL r4	0.8 μL B3<br>4 μL b3, 4 μL b6
